# Intra- and Interspecific Foraging and Feeding Interactions in Three Sea Stars and a Gastropod from the Deep Sea

**DOI:** 10.3390/biology12060774

**Published:** 2023-05-26

**Authors:** Brittney Stuckless, Jean-François Hamel, Jacopo Aguzzi, Annie Mercier

**Affiliations:** 1 Department of Ocean Sciences, Memorial University, St. John’s, NL A1C 5S7, Canada; bstuckless@mun.ca; 2 Society for the Exploration and Valuing of the Environment (SEVE), Portugal Cove-St. Philip’s, NL A1M 2B7, Canada; jfhamel.seve@gmail.com; 3 Instituto de Ciencias del Mar (ICM-CSIC), Paseo Marítimo de la Barceloneta, 08012 Barcelona, Spain; jaguzzi@icm.csic.es; 4 Zoological Station, Anton Dohrn (SZN), Villa Comunale, 80121 Naples, Italy

**Keywords:** competition, deep sea, behaviour, species interactions, gastropod, sea star

## Abstract

**Simple Summary:**

Competitive interactions among animals come in a variety of forms and may be influenced by the size and number of the individuals involved, and whether these individuals are from the same species or not. The deep sea is a food-limited environment where it can be assumed that larger or faster scavengers might have an advantage over smaller or slower ones. However, very little work on competitive relationships in deep-sea megabenthic species has been done. This study looked at four co-existing deep-sea invertebrate species of the Northwest Atlantic using time-lapse video recordings in controlled, darkened laboratory experiments. We measured food approach and consumption dynamics in interspecific and intraspecific trials with individuals of similar and differing sizes. Diverse competitive and cooperative behaviours occurred depending on the species, relative body sizes, and the number of individuals present. Surprisingly, larger or faster individuals (or species) did not always outcompete smaller or slower ones. In addition, some species changed their foraging based on competitive pressure, while others did not. Overall, this study sheds light on the feeding strategies of co-existing deep-sea invertebrates and the behavioural relations among them, providing important baseline ecological information for the comprehension of food web structures in remote and poorly understood environments.

**Abstract:**

Competitive interactions come in a variety of forms and may be modulated by the size and number of individuals involved, and/or the resources available. Here, intra- and interspecific competitive behaviours for food (i.e., foraging/food search and feeding/food ingestion) were experimentally characterized and quantified in four co-existing deep-sea benthic species. Three sea stars (*Ceramaster granularis*, *Hippasteria phrygiana*, and *Henricia lisa*) and one gastropod (*Buccinum scalariforme*) from the bathyal Northwest Atlantic were investigated using video trials in darkened laboratory conditions. A range of competitive or cooperative behaviours occurred, depending on species (conspecific or heterospecific), comparative body size, and the number of individuals involved. Contrary to expectations, small individuals (or smaller species) were not always outcompeted by larger individuals (or larger species) when foraging and feeding. Moreover, faster species did not always outcompete slower ones while scavenging. Overall, this study sheds new light on scavenging strategies of co-existing deep-sea benthic species in food-limited bathyal environments, based on complex behavioural inter- and intraspecific relationships.

## 1. Introduction

Intraspecific and interspecific interactions such as fighting [1], food stealing [2], or competitive exploitation (one individual using a resource before the other can; [3,4]) can have a profound impact on the feeding behaviours and ingestion rates of many marine benthic species [5,6]. Individual traits related to body size and physiology (e.g., hunger state) can modulate foraging efficiency and competitive ability in various marine invertebrate taxa such as crustaceans [7,8], gastropods [4], and echinoderms [9,10,11].

Studies of competitive interactions among sea stars and gastropods have so far centred on shallow-water ecosystems. In nearshore rocky environments, sea stars are frequently described as keystone predators, primarily feeding upon molluscs and occasionally on other echinoderms [5,12,13,14,15]. Menge and Menge [3] observed aggressive feeding interactions, such as pinching with the pedicellariae, between the sea stars *Pisaster ochraceus* and *Leptasterias hexactis*, with the presence of the former reducing the time the latter spent foraging. Similarly, Rogers et al. [11] found evidence of *P. ochraceus* being aggressive and dominant over the co-existing sea star *Evasterias troschelii*, inducing avoidance behaviours in the latter. Furthermore, St-Pierre et al. [6] reported that foraging (as food search) and feeding (as food ingestion) of the sea star *Asterias rubens* can be altered by the presence of the crab *Cancer irroratus*, suggesting that the identity of a competitor affects food-related behaviour in some species.

Gale et al. [16] and Stuckless et al. [17] recorded specificity in diets and feeding strategies among bathyal co-existing sea stars. Collectively, their results showed that *Ceramaster granularis* and *Henricia lisa* are sponge eaters that can also scavenge various types of carrion, while *Hippasteria phrygiana* is a prominent predator of cnidarians with detritivorous tendencies. However, intra- and interspecific interactions during food search and ingestion remain unexplored, and whether deep-sea asteroids display similar competitive dynamics as their shallow-water counterparts is an understudied topic.

Gastropods of the genus *Buccinum* are both predators and scavengers, targeting polychaetes, bivalves, and sea urchins, and scavenging upon various species of fishes [18,19,20]. In addition, some species display opportunistic kleptoparasitism (stealing food; [2,21]). Gravid females of *Buccinum undatum* are more likely to kleptoparasitize the food of the sea star *Lepasterias polaris* than males, potentially due to the reproductive benefits of securing extra resources, despite the predation risk the sea star poses [2]. However, no studies to date have examined the potential interactions of *Buccinum scalariforme* in the context of inter- and intraspecific competition for food resources.

In the present study, we investigated how four co-existing deep-sea species, three sea stars (*C. granularis*, *H. phrygiana*, and *H. lisa*) and one gastropod (*B. scalariforme*), responded to various forms of intra- and interspecific competition for food. This study, which explores interactions among two or more individuals, builds upon the methodology of an earlier study by Stuckless et al. [17], which examined singleton responses of these four species. Here we describe and then quantify the behaviour that occurred among conspecifics of comparable or different sizes and among the four different species. More precisely, we tested if larger individuals would always outcompete smaller conspecifics (e.g., either excluding an individual from feeding, dislodging an individual part-way through feeding, or forcing an individual to relocate on food to accommodate the new arrival), and whether faster-moving species would systematically outcompete slower ones for access to sporadic and limited carrion resources.

## 2. Materials and Methods

### 2.1. Focal Species, Collection, and Holding Conditions

The four focal species co-exist and are common in the deep waters of the Northwest Atlantic. The sea star *C. granularis* inhabits depths ranging from ~50 to >1400 m in the Arctic Ocean and on both sides of the North Atlantic Ocean, to as far south as the Northeast coast of South America [22,23]. The sea star *H. phrygiana* is found throughout the Northern and Southern Atlantic and Pacific oceans at depths ranging between 10 and 1400 m [24]. The sea star *H. lisa* is reported in deep waters of the North Atlantic basin down to ~1400 m [25]. Finally, the gastropod *B. scalariforme* is found between subtidal and bathyal depths (> 1100 m) in the Arctic Ocean, along both coasts of Canada, off Greenland, off Iceland, and off Alaska and Maine in the USA [26].

Individuals of each species were collected as opportunistic trawl by-catch between 2013 and 2017, during routine surveys conducted by Fisheries and Oceans Canada (DFO) off the eastern coast of Newfoundland Canada between 800 and 1500 m depths. Collections took place in late autumn and early winter, at a time when surface temperatures were within tolerable ranges for deep-sea species (typically between 1 °C and 6 °C). Holding mesocosms had a ~15–20 cm thick layer of muddy substrate available, along with some rocks and deep-sea corals. All individuals were acclimated to the laboratory mesocosms (several months) before using them for experiments and only healthy individuals (i.e., determined to be responsive, active, and without any visible injuries) were chosen. Further details of collection and maintenance are provided in Stuckless et al. [17].

### 2.2. Experimental Designs

The initial response of the four focal species to the presence of food was examined in a series of eight separate, short-term experiments (≤77 min) in which the presence of conspecific and/or heterospecific individuals, and the size of focal individuals, were manipulated (see Table S1). Four experiments, one for each species, involved placing two conspecific individuals of similar size (<25% difference in diameter/length, Table S1) in a single tank and an additional two experiments—one for *C. granularis* and the other for *H. phrygiana*, which had a larger range of sizes available—used two conspecific individuals ≥25% difference in diameter (Table S1). The final two experiments involved a heterospecific mixture of two species that fed upon the same food types in Stuckless et al. [17], one with *C. granularis* X *H. lisa*, and the other with *C. granularis* X *B. scalariforme*. Four trials were run for each experiment with food present, and four trials were run without food present (as a control). Trials were ended when the focal individuals no longer showed interest in the presence of food or stopped moving. The specific conditions for ending a trial were the same as in Stuckless et al. [17]. The time taken for individuals to reach a food item and to initiate feeding behaviours, as well as the path individuals took over the course of the trial, was recorded along with any other visible interactions and behaviours of note (e.g., individuals making physical contact, retraction of terminal podia or siphon). 

Five longer experiments (18–23 h) were conducted to examine behaviours among different individuals at a site with food (e.g., removal of feeding individuals by newly approaching ones or potential defensive behaviours of already-feeding individuals to avoid removal; Table S1). Two of the experiments—one for *C. granularis* and the other for *H. phrygiana*—used five conspecifics of different sizes placed in a single tank. These experiments had four trials each. In the third, fourth, and fifth experiments a heterospecific mixture was used as follows: *C. granularis* X *H. lisa*; *C. granularis* X *B. scalariforme*; and finally, *C. granularis* X *H. lisa* X *B. scalariforme*. The experiments with heterospecific pairs had two trials each (due to limited food resources available to the researchers) and the heterospecific triad had four trials.

### 2.3. Experimental Conditions and Procedures

Trials were conducted in 320 L tanks (80 cm wide × 126 cm long × 29 cm deep). Experimental tanks had no substratum as all focal species had been observed indiscriminately occupying all hard substrata including tank surfaces in the holding mesocosms [17] and the addition of substratum into the experimental tanks would introduce uncontrolled variables. The omission of natural substratum is common in similar types of studies [10,20]. 

The activity of individuals was recorded in time-lapse mode with an infrared camera (Brinno TLC 200 Pro and MAC 119 200 DN; Taipei City 11493, Taiwan) that acquired one picture per 30 s with a field of view encompassing the entire tank. An additional camera (Brinno TLC 200 Pro) was placed on the side of the tank near the food item and acquired one picture per 10 s, in order to more closely focus on the interactions with and around food stimuli. Images were analysed one by one using the ImageJ plug-in MtrackJ [27]. 

All replicates of a given experiment were run within four weeks, and no individual was used twice in the span of 48 h. All individuals were fasted for a minimum of two weeks prior to experimental trials. Individuals were placed 1–3 cm apart and equidistant from the food source. Details of cleaning and setting up tanks for each trial, the positioning of the focal individuals within a tank at the start of a trial, and the handling of data can be found in Stuckless et al. [17], which used identical procedures but with only one individual per tank. The deep-sea species used as food stimuli are found in the same habitat as the focal species and were based on the results of a previous study using the same four focal species [17] and mesocosm observations. The following species were used: the deep-sea octopus *Graneledone verrucosa* (tentacles), the deep-sea cup coral *Flabellum alabastrum* (whole), and fragments of deep-sea sponges (*Mycale lingua*, *Iophon* sp., and *Asconema* sp.). Food items used in the different trials are listed in Table S1.

### 2.4. Morphometric Measurements

All individuals were measured while submerged after the completion of each trial, minimizing the influence of handling stress on experimental results. Sea stars were measured haphazardly in three arms from central disk to arm tip; the mean of these three values was used to determine the diameter of each individual. For gastropods, the length of the shell was measured from the apex to the bottom edge of the aperture. These measurements were used to quantify the sizes of individuals used in all experiments, create size classes for *C. granularis* and *H. phrygiana*, and to confirm that individuals used in experiments met the size difference threshold where applicable (described in Section 2.2). 

Individuals of *C. granularis* were separated into three size classes: small (3.7 ± 0.2 cm), medium (4.7 ± 1.1 cm), and large (6.9 ± 0.2 cm). Individuals of *H. phrygiana* were also separated into three size classes: small (11.9 ± 0.3 cm), medium (15.3 ± 2.3 cm), and large (17.9 ± 1.3 cm). Individuals of *H. lisa* were all of similar size (5.3 ± 1.0 cm) and individuals of *B. scalariforme* were all of similar shell length (6.5 ± 0.7 cm). The complete list of size classes used in the various experiments is available in Table S1.

Additional measurements were used to better assess how physical traits may have influenced the results. A subset of individuals from all four focal species was used to assess the relationship between size and wet weight (Figure S1) and photos were taken of the oral side of the weighed sea stars for further measurements with ImageJ. The area (cm^2^) of the oral opening was measured (from the centre of the mouth to the first ambulacral podia), along with the area covered by the ambulacral groove (proxy for the area of aboral surface covered by podia) and the rest of the aboral surface. From these measurements, a percent area covered by ambulacral groove (i.e., podia) was calculated for each individual and then averaged for each sea star species (Table S2).

### 2.5. Response Variables and Data Processing

Short trials were analysed frame by frame (i.e., 30 s time interval; see Section 2.3) using the ImageJ plug-in MtrackJ [27] to determine mean and maximum speed and total distance travelled for every individual, as well as every 10 frames (5 min intervals) to determine the average change in path angle for individuals that interacted with the food. Paths with an average change in angle between points of ≤25° were considered straight, while paths with an average change in angle between points of >25° were considered curved. The reference point between frames to measure movement was the oral area in all species, i.e., the centre of the disk in sea stars, and the head in gastropods. Responses were scored as positive or negative (feeding vs. no feeding attempt), while all control responses were analysed together. For all trials any other additional behaviour of note (e.g., pushing, climbing over each other, etc.) was also recorded. 

For prolonged trials, videos were analysed to determine the dominant behaviour of each individual every hour, and the proportion of time each of them spent engaging in the different categories of behaviour: immobile (≤1 body length of movement); mobile (>1 body length of movement); inter-individual contact (touching any other individual); contact with stimulus (food or control item); feeding (adopting feeding postures/behaviours); and finally, unknown (when the animal was not visible due to being obscured by the drainage/inflow pipes or another animal). The results of this hourly analysis are available in Table S3.

### 2.6. Data Analysis

All values were presented as mean ± standard deviation (SD) where multiple data points were available, except for response times, which were provided as median ± SD. One-way analysis of variance (ANOVA) with post-hoc Tukey tests were run to compare responses (positive, negative, and control) for distance travelled, mean speed, and maximum speed. If data were non-normal or had unequal variance, a Kruskal–Wallis with post-hoc Dunn’s test was used instead. Two-way ANOVAs with post-hoc Tukey tests were run for trials involving two individuals of differing sizes of *C. granularis* and *H. phrygiana*, to determine if small and large classes along with a positive, negative, or control response had any effect on distance travelled, and mean or maximum speed. Two-way ANOVAs with post-hoc Tukey tests were also used for short-duration trials involving two different species, to determine if response and/or species affected distance travelled, mean speed, or maximum speed, unless there were uneven response types (i.e., one species had positive responses but the other did not), in which case unpaired *t*-tests were used. Where a significant interaction between factors occurred (i.e., species and response type), one-way ANOVAs with post-hoc Tukey tests or unpaired *t*-tests were used, as appropriate, within factors. All tests used a significance value of *p* < 0.05, however, results were interpreted cautiously, following calls to move away from arbitrary thresholds (including by the American Statistical Association; [28]), based on the principles of statistical clarity [29].

## 3. Results

### 3.1. Interspecific Interactions

Results in each of the species-specific sections below are outlined in the following sequence: effect of trial conditions and of response on the measured variables (i.e., paths, response time, distance travelled, and speed), behavioural interactions during foraging (i.e., searching for food), and behavioural interactions during feeding (i.e., actively consuming food) if any visible interactions occurred.

#### 3.1.1. The Sea Star *Ceramaster granularis*

In all treatments, regardless of the sizes and densities of the sea stars, individuals usually approached food in straight paths, led by the arm closest to the food. In trials with five individuals (where paths were more complex), that leading arm could change before approaching food, i.e., when an individual moved into the path of a larger conspecific that was heading for the food and required a change of orientation (see more detailed description below). Individuals positively responded to the food source in 21.0 ± 10.0 min for sea stars of similar sizes (*n* = 2), 40.0 ± 8.3 min for two differing sizes (*n* = 3), and 45.0 ± 150.8 min for five varying sizes together (*n* = 10; Table 1). 

**Figure 1:**
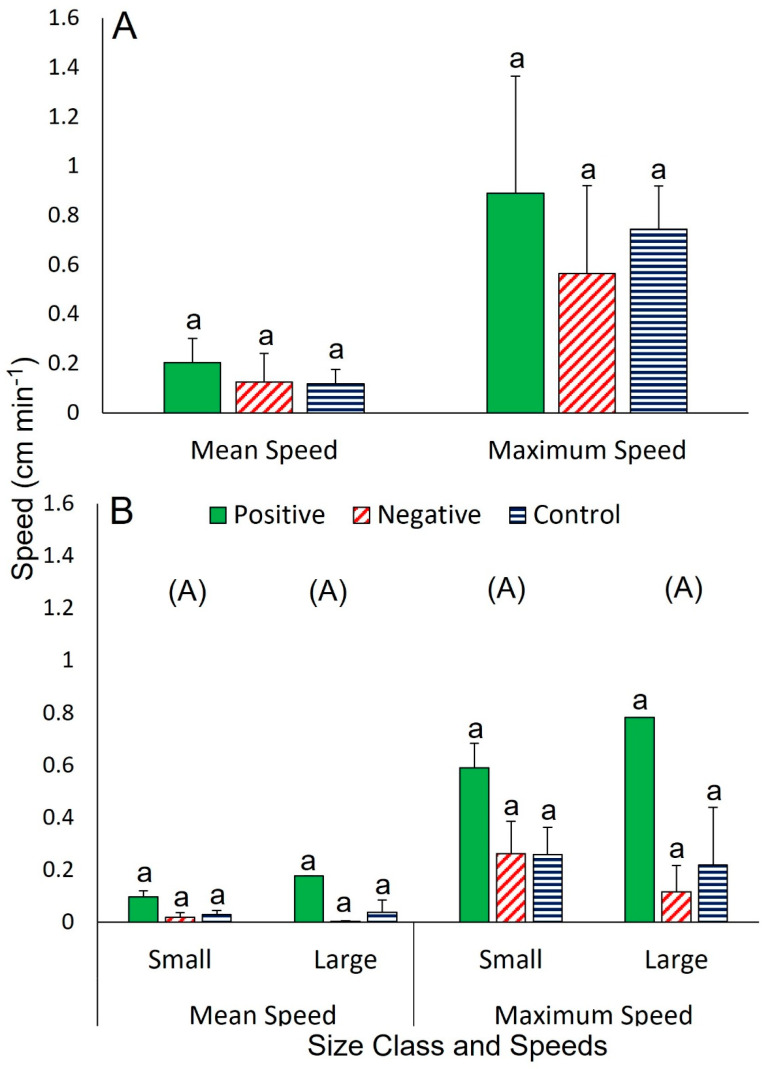
Mean and maximum speeds of *Ceramaster granularis* (**A**) when two similarly sized individuals were tested and (**B**) when two differently sized individuals were tested. Each of the two treatments was replicated four times and data are provided as means ± SD where applicable (*n* = 1–8). Means were compared between response types in one-way ANOVA with post-hoc Tukey test (*p* < 0.05) in A. Means were compared between sizes for each metric in B using two-way ANOVA with post-hoc Tukey test (*p* < 0.05). Bars with different letters are significantly different and groups with different letters are significantly different (within the same metric). See text for full results.

Individuals that did not approach the food, and individuals in the controls, were mostly stationary but when they moved, no clear leading arm preferences were noted (i.e., not rotating to keep a specific arm in the lead; Table S4). No difference in distance travelled was detected among sea stars of similar size during positive, negative, or control responses (averaging 5.8–9.0 cm; one-way ANOVA, *F*_2,11_ = 0.51, *p* = 0.612). Similarly, there was no difference either in mean speed (averaging < 0.1–0.2 cm min^−1^, one-way ANOVA, *F*_2,13_ = 0.93, *p* = 0.424), or maximum speed (averaging 0.13–0.89 cm min^−1^; one-way ANOVA, *F*_2,13_ = 1.07, *p* = 0.378; Figure 1A). In trials involving two sea stars of different size, neither class (large or small) nor response (positive, negative, or control) affected distance travelled (varying widely from 0.1 to 7.6 cm; two-way ANOVA; *F*_1,19 and 2,18_ = 0.94 and 2.46, *p* = 0.121 and 0.349, respectively), mean speed (0–0.2 cm min^−1^; two-way ANOVA, *F*_1,19 and 2,18_ = 0.50 and 1.93, *p* = 0.491 and 0.18), or maximum speed (0.1–0.8 cm min^−1^; two-way ANOVA, *F*_1,19 and 2,18_ = 2.99 and 1.00, *p* = 0.106 and 0.392).

Behavioural interactions during foraging varied depending on the size and number of conspecifics present. As the number of conspecifics increased from 2 to 5, so did the number of total inter-individual contacts (from 0.5 ± 0.5 to 2.8 ± 1.3 contacts per trial) despite ample space being available in the tank for avoidance. When 2 individuals of similar size foraged together, they maintained their distance with each other (>1 cm) with only brief moments of contact. In two of the trials (50%) involving 5 individuals of varying sizes foraging together, a small or medium-sized individual moved into the path of a larger one that was heading for the food. This occurred at 11 and 20 min from the start of the trial and this “interception” lasted 3 min and 16 min, respectively. The large individual eventually abandoned its trajectory toward the food and moved away, while the small or medium sea stars approached the food. The intercepting individual then joined the others at the food (Video S1). 

Behavioural interactions during feeding varied depending on the size of the already-feeding individual(s). In one instance (25% of trials with two individuals), a large individual reached the food first and a smaller individual (arriving second) attempted to push itself under its body (a behaviour we have called wedging; Video S2) to access the food and enable both individuals to feed at the same time. In a reverse scenario (i.e., a large individual arriving after a smaller one) the larger individual pushed the smaller away from the food (once, 25% of trials with 5 individuals, Video S3). A behavioural interaction occurred between small or medium feeders when individuals touched reciprocally via arm tips for 13–16 min; then their positions changed so the arriving individual could also access the food. Up to 4 individuals were seen moving from their previous positions to allow all conspecifics to access the food (50% of trials with 5 individuals; Video S1). 

#### 3.1.2. The Sea Star *Hippasteria phrygiana*

**Figure 2:**
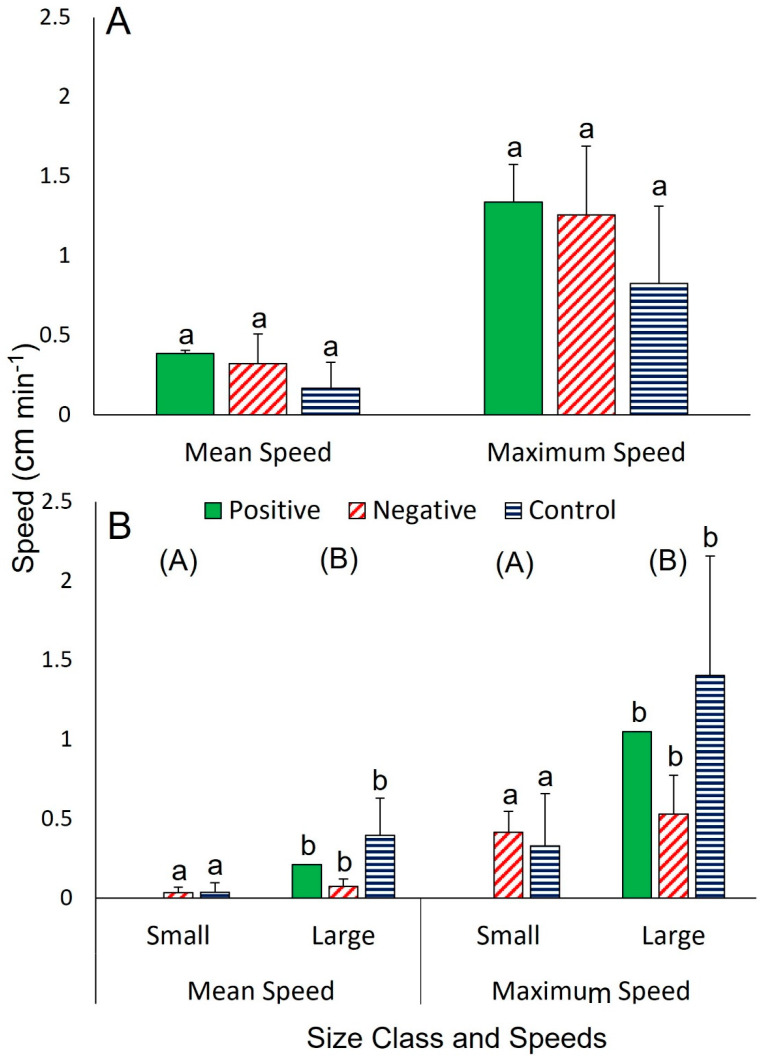
Mean and maximum speeds of *Hippasteria phrygiana* (**A**) when two similarly sized individuals were tested and (**B**) when two differently sized individuals were tested. Each of the two treatments was replicated four times and data are provided as means ± SD where applicable (*n* = 1–8). Means were compared between response types in one-way ANOVA with post-hoc Tukey test (*p* < 0.05) in A. Means were compared between sizes for each metric in B using two-way ANOVA with post-hoc Tukey test (*p* < 0.05). Bars with different lower letters are significantly different and groups with different capital letters are significantly different (within the same metric). See text for full results.

In all treatments, individuals approached food in straight paths unless they encountered a conspecific, in which case they skirted around each other, only brushing arm tips, rather than crawling over each other (Video S4). Positive responses to the food occurred in 35.5 ± 11.3 min (*n* = 4) in trials involving two similarly sized individuals, in 58 min (*n* = 1) involving a pair of unequal sizes (i.e., one larger and one smaller), and in 50.0 ± 444.1 min (*n* = 5) in trials involving 5 individuals of various sizes (Table 1). In the absence of a response to the food, or in the controls, individuals either remained stationary or travelled around the tank without clear direction (Figure 2, Table S4). In trials with similarly sized sea stars, no clear differences were detected between positive (*n* = 4), negative (*n* = 4), or control responses (*n* = 8) for distance travelled (10.5–22.7 cm; one-way ANOVA, *F*_2,13_ = 2.13, *p* = 0.159), mean speed (0.17–0.4 cm min^−1^; Kruskal-Wallis test, *H*_2_ = 3.99, *p* = 0.159), or maximum speed (0.8–1.3 cm min^−1^; one-way ANOVA, *F*_2,13_ = 2.47, *p* = 0.123; Figure 2A). In trials involving two differently sized sea stars, size was found to have an impact on the absolute distance travelled (two-way ANOVA, *F*_1,19_ = 4.85, *p* = 0.044) while the influence of response type was less clear (two-way ANOVA, *F*_2,18_ = 2.83, *p* = 0.091) and there was no interaction between these two factors. There was a clear interaction between body size and response type for mean speed (two-way ANOVA, *F*_2,18_ = 5.42, *p* = 0.017); independent tests showed that response type (positive, negative, or control) did not affect mean speed for either size class (small: no positive responses, >0.1 cm min^−1^ for negative and control responses, *n* = 4 for both types; unpaired *t*-test, *t* = 0.1, *p* = 0.924; large: 0.2–0.4 cm min^−1^ and *n* = 1, 3, 4, respectively; one-way ANOVA, *F*_2,5_ = 2.57, *p* = 0.17). However, mean speed was statistically different between size classes, with large individuals (*n* = 8) traveling faster than small ones (*n* = 8; large: 0.3 ± 0.1 cm min^−1^, small: 0.03 ± 0.02 cm min^−1^; unpaired *t*-test, *t* = 2.62, *p* = 0.02; Figure 2B). There was also an interaction between size and response type for maximum speed (two-way ANOVA, *F*_2,18_ = 3.93, *p* = 0.042) and independent tests showed that response type (positive, negative, or control) did not clearly affect maximum speed for either size class (small: no positive responses, 0.4 ± 0.1 and 0.3 ± 0.3 cm min^−1^; unpaired *t*-test, *t* = 0.49, *p* = 0.641; large: 0.5–1.4 cm min^−1^; one-way ANOVA, *F*_2,5_ = 1.77, *p* = 0.263) but that, similar to mean speed, maximum speed clearly differed between size classes, with large individuals traveling faster (large: 1.0 ± 0.2 cm min^−1^, small: 0.4 ± 0.1 cm min^−1^; unpaired *t*-test, *t* = 2.67, *p* = 0.019; Figure 2B).

As conspecific density increased from 2 to 5 individuals, the number of interactions increased from 0.3 ± 0.5 to 3.0 ± 1.6 contacts per trial despite space being available in the tank for individuals to avoid contact with each other. When two similar-sized sea stars were tested, only one moved towards the food, while the other either disregarded the food and moved away (in 75% of trials) or remained stationary (25%). In the case of two individuals of different sizes, both remained stationary in 3 trials (75% of trials); in the remaining trial, the larger individual approached the food while brushing along the immobile smaller individual (~55 min of contact between individuals) before reaching the food and beginning to feed.

Food was always monopolized by a single individual, which was the first individual to reach the food in most cases. One exception was when the smallest sea star in a group of 5 conspecifics reached the food first; a medium-sized individual approached ~60 min later and partially overlapped the smaller conspecific. The interaction lasted for 36 min until the smaller individual retreated, leaving the larger sea star to access the food (Video S5).

#### 3.1.3. The Sea Star *Henricia lisa*

**Figure 3:**
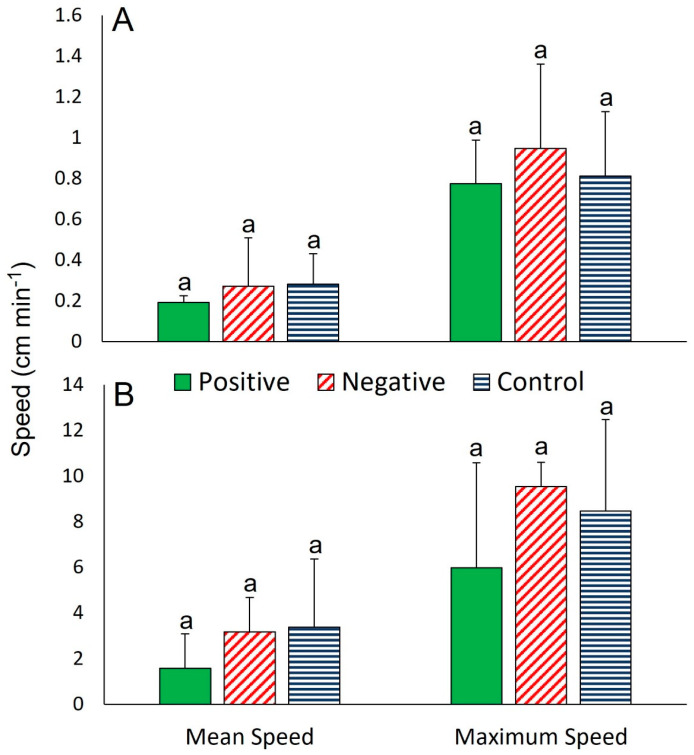
Mean and maximum speeds of (**A**) *Henricia lisa* and (**B**) *Buccinum scalariforme* when two individuals were tested concurrently. Each of the two treatments was replicated four times and data are shown as mean ± SD (*n* = 3–8). Means were tested across different response types for each metric using one-way ANOVA with post-hoc Tukey test (*p* < 0.05). Bars with different letters are significantly different from each other (for a given metric). Note the different Y-axis between panels. See text for full results.

When approaching food, paths were either straight (33%) with the arm closest to the food leading or curved (66%) where the lead arm was shifted from the original arm to a new arm that was closer to the food (Video S6). The response time of individuals reaching the food was 26.0 ± 22.5 min (*n* = 3; Table 1), whereas individuals that disregarded the food (*n* = 5) and individuals in the control treatment (*n* = 8) travelled around the tank without a clear direction (Table S4). In trials involving two individuals, there were no clear differences detected between positive (*n* = 3), negative (*n* = 5), or control (*n* = 8) response types for distance travelled (9.5–14.8 cm; one-way ANOVA, *F*_2,13_ = 0.33, *p* = 0.723), mean speed (0.2–0.3 cm min^−1^; one-way ANOVA, *F*_2,13_ = 0.34, *p* = 0.722) or maximum speed (0.8–1.0 cm min^−1^; one-way ANOVA, *F*_2,13_ = 0.31, *p* = 0.742; Figure 3A, Table S4).

When two individuals of similar size were tested together, they made contact (about 20 min) through the podia at the tip of their arms in 50% of trials (1.0 ± 1.4 contacts per trial). When reaching the food, in 66% of all positive trials, individuals displayed “tapping” behaviour (the tip of an arm was successively applied to and lifted from the food; ~3 min between taps, ≥ 3 taps per observation) with limited movement of the arm tip (~1 cm displacement per tap) over the food (Video S7). 

#### 3.1.4. The Gastropod *Buccinum scalariforme*

Paths toward the food were either straight from the onset (66% of positive trials; Video S8) or haphazard in the remaining cases, lasting for ~45 min and then becoming straight upon approaching food (33% of positive trials). The response time was 3.0 ± 22.8 min (*n* = 3, Table 1). Individuals that disregarded the food (*n* = 5) or that were in the control treatment (*n* = 8) travelled around the tank without clear direction (Table S4). In trials involving two individuals at the same time, no clear differences were detected between positive (*n* = 3), negative (*n* = 5), or control (*n* = 8) response types for distance travelled (67.5–104.3 cm; one-way ANOVA, *F*_2,13_ = 0.18, *p* = 0.834), mean speed (1.6–3.4 cm min^−1^; one-way ANOVA, *F*_2,13_ = 0.99, *p* = 0.399), or maximum speed (6.0–9.6 cm min^−1^; one-way ANOVA, *F*_2,13_ = 0.63, *p* = 0.550; Figure 3B, Table S4).

Contact among paired conspecifics occurred only once across all four trials (0.3 ± 0.5 contacts), wherein the siphon of one individual touched the shell of the other for < 10 s. When an individual moved toward and touched the food (75% of trials, one individual per trial), the siphon continuously made sweeping movements and remained pointed in the direction of the food item.

### 3.2. Interspecific Interactions

#### 3.2.1. The Sea Star *C. granularis* with the Sea Star *H. lisa*

**Figure 4:**
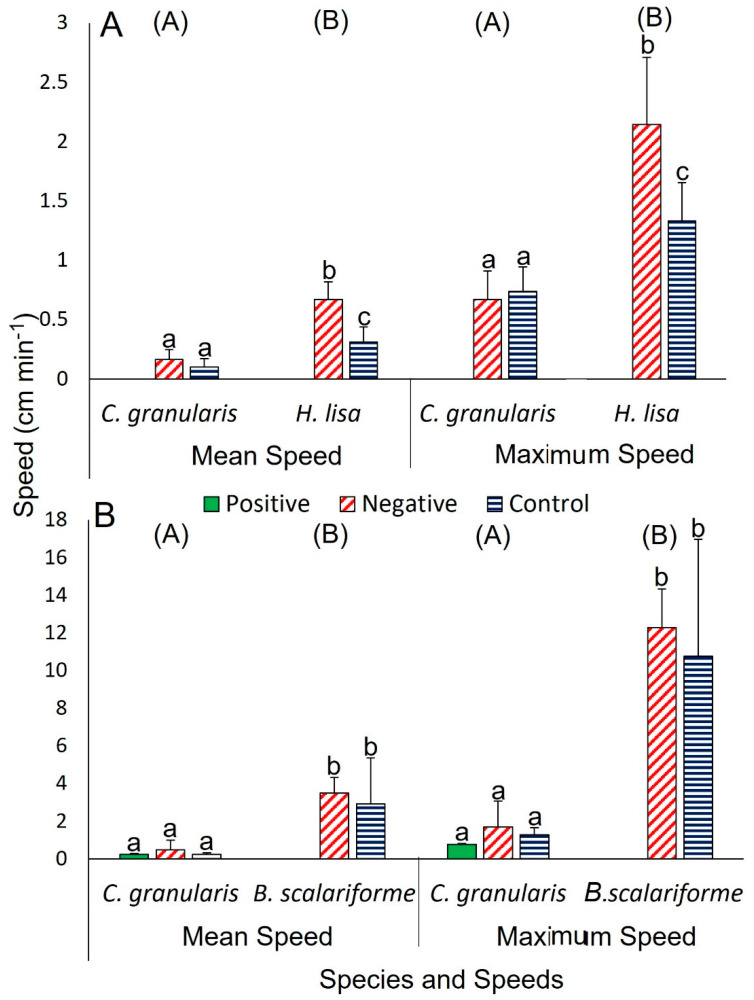
Mean and maximum speeds for (**A**) *Ceramaster granularis* and *Henricia lisa* tested concurrently and (**B**) *C. granularis* and *Buccinum scalariforme* tested concurrently in short-duration trials. Please note different Y-axis scales for A and B. Each of the two treatments was replicated four times and data are shown as mean ± SD (*n* = 2–8). Means were tested within species and between species using two-way ANOVA in panel A and one-way ANOVA with post-hoc Tukey tests and *t*-tests for each factor as appropriate in panel B due to *B. scalariforme* having no positive trials. Bars with different letters are significantly different within a species for a given metric while groups with different letters are significantly different between species (*p* < 0.05). See text for full results.

In short trials, distance travelled was affected by species (two-way ANOVA, *F*_1,14_ = 18.58, *p* = 0.001) and response type (two-way ANOVA, *F*_1,14_ = 7.71, *p* = 0.017) without any interactions. Specifically, *H. lisa* clearly travelled further during negative responses (42.1 ± 17.8 cm, *n* = 4) than during control responses (18.0 ± 6.1 cm, *n* = 4; unpaired *t*-test, *t* = 2.57, *p* = 0.04 Table S4) and there were no positive responses. There was an interaction between species and response type for mean speed (two-way ANOVA, *F*_1,14_ = 6.97, *p* = 0.023). Independent tests showed no clear effect of response type (negative or control) on mean speed for *C. granularis* (0.2–0.1 cm min^−1^, *n* = 4 for both; unpaired *t*-test, *t* = 1.19, *p* = 0.279), while response type did affect the mean speed of *H. lisa*, with individuals in negative trials traveling faster than those in control trials (0.7 ± 0.2 and 0.3 ± 0.1 cm min^−1^ *n* = 4 for both; unpaired *t*-test, *t* = 3.69, *p* = 0.01). There was a difference between species, with *H. lisa* traveling faster than *C. granularis* (*H. lisa*: 0.5 ± 0.1 cm min^−1^ and *C. granularis*: 0.1 ± 0.03 cm min^−1^, *n* = 8 for both; unpaired *t*-test, *t* = 4.15, *p* = 0.001; Figure 4A). There was also a clear interaction between factors (species and response type) for maximum speed (two-way ANOVA, *F*_1,14_ = 5.91, *p* = 0.032); independent tests showed that response type influenced the maximum speed of *H. lisa*, again with individuals in negative trials traveling faster than those in control trials (2.2 ± 0.6 and 1.3 ± 0.3 cm min^−1^, respectively, with no positive responses; unpaired *t*-test, *t* = 2.50, *p* = 0.047), but not *C. granularis* (0.7 ± 0.2 cm min^−1^ for both negative and control trials, with no positive responses; unpaired *t*-test, *t* = 0.42, *p* = 0.69). Similar to mean speed, there was a clear difference in maximum speed between species, with *H. lisa* traveling fastest (*H. lisa*: 1.7 ± 0.2 cm min^−1^, *C. granularis*: 0.7 ± 0.1 cm min^−1^; unpaired *t*-test, *t* = 4.54, *p* < 0.001; Figure 4A).

When one individual of *C. granularis* and of *H. lisa* were tested together, contact happened once in each of the short (*n* = 4) and prolonged (*n* = 2) trials. It involved the arms of *H. lisa* moving over the top of the immobile *C. granularis*, which displayed no visible avoidance behaviour. In short trials, neither species fed, with individuals of both species traveling around the tank or remaining stationary rather than approaching the food. However, *H. lisa* did travel further and faster than *C. granularis* in both experimental and control treatments (Table S4). 

In prolonged trials, *H. lisa* approached food with straight paths (100%), responding in 12.0 ± 8.5 min (*n* = 2, Table 1), and fed in 100% of trials, always displaying the tapping behaviour (described above). *Ceramaster granularis* did not feed in the prolonged experimental trials.

#### 3.2.2. The Sea Star *C. granularis* with the Gastropod *B. scalariforme*

In both short and prolonged trials, *B. scalariforme* did not approach the food, but it travelled over greater distances and faster than *C. granularis* (Table S4). *Ceramaster granularis* approached the food in straight paths (100% of positive trials), with the arm closest to the food acting as the leading arm, and fed in 50% of short-duration trials and in 100% of prolonged trials. The response time of *C. granularis* was 31.0 ± 17.0 min (*n* = 2) in the short trials and 17.5 ± 0.7 min (*n* = 2) in the prolonged trials (Table 1). Individuals of *C. granularis* that did not approach the food in short trials (*n* = 2), or individuals in the control treatment (*n* = 4), were either largely immobile or travelled short distances (Table S4). Distance travelled was clearly different between species (unpaired *t*-test; *t* = 4.29, *p* < 0.001), with *B. scalariforme* (*n* = 8) traveling further than *C. granularis* (*n* = 8), whereas it was not clearly different between positive, negative, or control response types in either *C. granularis* (10.5–28.7, *n* = 2, 2, and 4, respectively; one-way ANOVA, *F*_2,5_ = 1.10, *p* = 0.403) or *B. scalariforme* (145.3–190.1 cm, *n* = 4 for both negative and control trials, no positive responses; unpaired *t*-test, *t* = 0.61, *p* = 0.564; Table S4). The two species also exhibited different mean speeds, with *B. scalariforme* moving faster (*B. scalariforme*: 3.2 ± 0.6 cm min^−1^, *C. granularis*: 0.3 ± 0.1 cm min^−1^; unpaired *t*-test, *t* = 4.75, *p* < 0.001). Response type (positive, negative, or control) did not have a clear effect on the mean speed of *C. granularis* (0.2–0.5cm min^−1^; one-way ANOVA, *F*_2,5_ = 0.82, *p* = 0.491) or *B. scalariforme* (no positive responses, 3.5 ± 0.8 and 2.9 ± 2.4 cm min^−1^; unpaired t-test, *t* = 0.50, *p* = 0.669; Figure 4B). Maximum speed was markedly different between the two species, with *B. scalariforme* moving faster (*B. scalariforme*: 11.5 ± 1.5 cm min^−1^, *C. granularis*: 1.3 ± 0.2 cm min^−1^; unpaired *t*-test, *t* = 6.58, *p* < 0.001); however, there was no clear change in maximum speeds between response types (positive, negative, or control) for *C. granularis* (0.8–1.7 cm min^−1^; one-way ANOVA, *F*_2,5_ = 0.92, *p* = 0.458) or *B. scalariforme* (no positive responses, 10.8–12.3 cm min^−1^; unpaired *t*-test, *t* = 0.47, *p* = 0.657; Figure 4B).

When one individual of *C. granularis* and of *B. scalariforme* were together direct contact was rare. Direct contact/interaction occurred only once (12.5% of trials, a short trial), where *B. scalariforme* approached the sea star and then crawled over it within 2 min (no visible reaction from *C. granularis* was noted). In all other trials (87.5%), both individuals travelled around the tank without physical contact with each other.

#### 3.2.3. The Sea Star *C. granularis* with the Sea *Star H. lisa* and the Gastropod *B. scalariforme*

Each of the three species approached food in three of four experimental trials. *Henricia lisa* approached food in a straight path (100% of positive responses) with the arm closest to the food leading. *Henricia lisa* was the first to reach food in 50% of trials (*n* = 2), fed in 75% of trials (*n* = 3), and had a response time of 65.0 ± 145.2 min (*n* = 3, Table 1). *Buccinum scalariforme* usually approached food after traveling around the tank; however, once it began to approach food, the path was straight (100% of positive trials) with the siphon pointed towards the food. *Buccinum scalariforme* approached food from both upstream and downstream directions; it reached food first in 50% of trials (*n* = 2), fed in 75% of trials (*n* = 3), and had a mean response time of 103.0 ± 79.4 min (*n* = 3, Table 1). *Ceramaster granularis* approached food in a straight path (100% of positive trials, *n* = 3) with the arm closest to the food leading; it was never the first to reach food but fed in 75% of trials (*n* = 3) and had a mean response time of 188.0 ± 341.7 min (*n* = 3; Table 1).

When one individual of *C. granularis*, of *H. lisa*, and of *B. scalariforme* were tested together, at least two of the three species made contact 2.3 ± 1.3 times per trial. In 75% of trials, *B. scalariforme* crawled over the other individuals (both sea star species); this contact was ~2 min and neither sea star species showed any visible reaction (e.g., moving away, arm curling, retraction of terminal podia). There was one instance of longer contact by *B. scalariforme* where it remained over the arm of *H. lisa* while both individuals were feeding (~4 h). *Ceramaster granularis* engaged in previously described wedging behaviour in 50% of trials (*n* = 2), attempting to push itself under the arms of *H. lisa* or under the foot of *B. scalariforme* to reach food (Video S9). Wedging resulted in *H. lisa* pulling its arms back and relocating on the food. Once *C. granularis* was established on the food, both species fed while still touching for the remainder of the trial. Wedging had mixed results against *B. scalariforme*. In one instance, *B. scalariforme* relocated to continue feeding, and in the other, *B. scalariforme* pushed back against *C. granularis*, forcing the sea star to move to an unoccupied area on the food.

## 4. Discussion

In the present study, we characterized intra- and interspecific interactions during foraging and feeding in four co-existing deep-sea species (the sea stars *Ceramaster granularis*, *Hippasteria phrygiana*, and *Henricia lisa*, and the gastropod *Buccinum scalariforme*). These interactions involved a range of competitive or cooperative behaviours, depending on species, body size, and number of individuals involved. While food supply is one of the main limiting factors in the deep sea [30,31,32], very few field or laboratory studies have examined food retrieval and ingestion behaviours in benthic megafauna [33,34,35], and to our best knowledge none exist in the context of dynamic interactions among co-existing species. 

Contrary to initial assumptions, small individuals of a species (or smaller species) were not always outcompeted by larger conspecifics (or larger species) for access to food. Moreover, faster species did not always have better access to food than slower species in scavenging trials. Specifically, *C. granularis* displayed a mix of agonistic and cooperative intraspecific interactions, *H. phrygiana* showed agonistic responses towards conspecifics, and *H. lisa* displayed no obvious intra- or interspecific interactions, remaining isolated. Surprisingly, the gastropod *B. scalariforme* exhibited little intraspecific interaction, despite the fact that shallow-water snails show a range of intraspecific behaviours including gregarious feeding [36,37,38] and kleptoparasitism [39].

Competition or cooperation as a flexible behaviour while foraging in sea stars was reported here for the first time, possibly based on reciprocal size assessment (potentially chemosensory based, see below). For example, when *C. granularis* was foraging with multiple conspecifics (i.e., a group of five individuals of varying sizes) small individuals displayed an intercepting behaviour by blocking the path of larger ones, allowing the other small conspecifics to reach the food first and to redistribute themselves around it, after which the small individual involved in the initial interception also moved to the food. In echinoderms of the class Holothuroidea (sea cucumbers), individual scent is sex- and size-specific [40]. A similar situation may exist in *C. granularis* explaining why when more than two individuals are present in a foraging/feeding scenario, individuals cooperated when equally sized, and competed when unevenly sized. This finding suggests that gregarious feeding among smaller individuals may prevent them from being excluded by larger ones, as seen in *H. phrygiana* (see below). In turn, this behaviour could have a detrimental effect on the feeding success of larger individuals by reducing their access to food when they are outnumbered by smaller conspecifics. 

In contrast, the sea star *H. phrygiana* displayed intraspecific agonistic behaviour by always attempting to monopolize food resources (e.g., solitary cup corals) with larger individuals dislodging smaller conspecifics from the food if smaller individuals reached the food first. This is the only clear example of size-asymmetric competition in this study, with larger individuals having a distinct competitive advantage over smaller ones when attempting to monopolize food resources. Higher aggressivity and competitivity in bigger individuals with larger pedicellariae (used for pinching) occurs in shallow-water species [5,11] and *H. phrygiana* has large pedicellariae on both the oral and aboral surfaces, possibly allowing for similar behavioural trends [24]. Unlike many sea stars that possess adherent podia to firmly grasp hard substrata or prey (mechanism described by Hennebert [41]), the podia of *H. phrygiana* are larger and seem to have comparatively limited adherence (personal observations). In addition, the rigid body of *H. phrygiana* appears to render smaller individuals more susceptible to being dislodged during competitive interactions, such as pushing or wedging by larger conspecifics.

*Henricia lisa* differed from the other sea stars (i.e., *C. granularis* and *H. phrygiana*) by showing little direct interaction among conspecifics. Encounters could be described as neutral (e.g., brushing of arms) and were infrequent. Tests with equally sized *P. ochraceus* and *E. troschelii* did not display inter- and intraspecific interference behaviours [11], aligning with our results. This lack of agonistic behaviour may also be explained because *H. lisa* feeds primarily upon sponges [17,35], which are large, sessile, and abundant in deep waters off the coast of Newfoundland [42,43]; therefore, intraspecific competition for this resource may be reduced. 

The fourth species studied was the gastropod mollusc *B. scalariforme* where interactions between similar-sized conspecifics were neutral and infrequent, similar to *H. lisa*. Field observations have reported deep-sea gastropods of varying sizes scavenging on the same food source simultaneously, with no signs of agonistic interactions [36], and the same appears to be true of laboratory trials in more controlled settings. 

Competitive interactions of heterospecific trials were detected in *C. granularis* with both *H. lisa* and *B. scalariforme*. The two sea star species may prey upon the same sponge species in the field [16,17], explaining their competitive interactions in limited-resource experiments. *Henricia lisa* was consistently outcompeted by *C. granularis* in trials involving pieces of octopus, as the latter wedged under the former and pushed it away. However, the rigid body wall of *C. granularis* may not allow it to access food located in complex environments (e.g., cervices, under rocks) as easily as the more flexible *H. lisa*. This suggests that these two species may exhibit distinct foraging strategies in nature and occupy different ecological niches in overlapping distribution areas. *Ceramaster granularis* is the slowest-moving of the four studied species [17], likely because of a combination of webbed arms and reduced podia coverage of the oral surface compared to *H. phrygiana* and *H. lisa* (20% vs. 25% and 29% podia coverage, respectively; Table S2). In contrast, webbed arms and a rigid body wall may provide a competitive advantage to *C. granularis* via the wedging strategy, thus diminishing the need to reach food quickly. By comparison, the long, narrow, and more podia-covered arms of *H. lisa* (29% vs. 20–25% coverage in *C. granularis* and *H. phrygiana*, respectively; Table S2), may explain the proportionally high speeds achieved by this species for its size [17]. This morphology could be advantageous when feeding upon the uneven and perforated surfaces of sponges that constitute common food sources for members of the *Henricia* genus [17,35,44]. However, such thin arms seem poorly suited to repelling competitors or tightly gripping smooth surfaces such as octopus tentacles. In contrast, when *B. scalariforme* was competing against *C. granularis* at a food source the former was sometimes able to use its muscular foot to repel wedging attempts by the latter, enabling it to compete against *C. granularis* more effectively for limited food resources than *H. lisa*. Other species of *Buccinum* have been observed to successfully compete for food with a wide variety of taxa, including sea stars, crabs, and fishes [2,21,36].

The present study allowed an exploration of whether the conspecific and heterospecific interactions discussed above modulate feeding by comparing the present results of multi-individual trials to those of Stuckless et al. [17], who used the same species in singleton trials. The time required by *C. granularis* to reach food remained consistent, with the exception of trials simultaneously involving both *H. lisa* and *B. scalariforme*, which elicited longer median response times. Prior works suggest that *C. granularis* is an opportunistic generalist [16,17], which may explain why this species responds to food cues relatively consistently with the exception of more complex heterospecific trials with two other competing species.

In singleton trials with *H. phrygiana*, Stuckless et al. [17] recorded variability in response times that was suspected to be driven by differing food stimuli, as supported by Gale et al. [16], suggesting that food types had differential palatability (and hence attraction potential). Consistent reaction times were observed when individuals were offered the same food (e.g., the cup coral *F. alabastrum*, which seems to be a preferred food for this species), further suggesting that the food type is the main driving factor in response time, as opposed to the presence or absence of competition. 

In contrast, *H. lisa* responded more quickly when intra- or interspecific competition was present than when foraging alone, which may be an indication of competition-based behavioural alteration. In half of the singleton trials by Stuckless et al., [17], individuals travelled in large loops when foraging; here, a straighter path was consistently detected. Pre-feeding assessment (e.g., longer cross-current loops in singleton trials to scan for odor plumes) may be diminished when competitors are present in order to prioritize faster consumption, similar to how the shallow-water *A. rubens* alters its foraging strategy when crabs are present in order to prioritize feeding [6]. 

Social interactions among sea stars are poorly known, so our results are difficult to compare with published works. However, there is mounting evidence that the social behaviour of sea stars may be more complex than currently assumed. The deep-sea species *Novodinia americana* is suspected to communicate via bioluminescence through its well-developed eyes [45] while the shallow-water species *Leptasterias polaris* uses chemical communication and physical contact to coordinate spawning events [46]. The results of our study, which suggest the capability for cooperation in some species, in conjunction with evidence from previous works suggesting complex social behaviours, indicate that the social capabilities of sea stars warrant further study to better understand their multiple ecological roles.

In contrast to the sea stars, the gastropod *B. scalariforme* responded more slowly to food cues in the presence of multiple individuals than when alone [17]. Individuals in trials would either directly approach the food (resulting in short response times) or would reach it after traveling haphazardly around the tank and then directly approaching food (resulting in long response times). This behavioural plasticity suggests the presence of intra- or interspecific competitors does not determine the food approach. Behavioural plasticity has been described in many other marine gastropods in regard to various stimuli, such as type of bait [20], predator presence [47], water acidity [48], and wave action [49].

## 5. Conclusions

The present study found variable competitive and cooperative behaviours in co-existing deep-sea benthic invertebrates, depending on the species, size, and number of the individuals involved. The initial hypothesis that larger individuals would always outcompete smaller individuals was not verified, as exemplified by what seems to be cooperative interference behaviour among small/medium-sized *C. granularis* against large conspecifics. Likewise, the hypothesis that faster species would consistently outcompete slower ones was challenged, as the slowest species in this study (*C. granularis*) was often able to access food even when set against faster species (*H. lisa* and *B. scalariforme*). *Henricia lisa* changed its foraging behaviour from longer, cross-current looping paths to shorter, direct paths when isolated or grouped, respectively, to approach the food more quickly when intra- or interspecific competitors were present. 

Understanding how animals interact intra- and interspecifically in their environment during foraging and feeding processes provides insights into potential dominance hierarchies and competitive dynamics [4,8,14,50,51]. As resource exploitation and other anthropogenic pressures in the deep sea (e.g., habitat loss or fishery discards) are likely to increase in the future, understanding how currently understudied deep-water ecosystems function, and how the species within them interact, should allow us to better protect these unique areas and species [52,53,54,55]. 

## Figures and Tables

**Table 1:** Median response time (time to reach food as min ± SD; *n* = 1–25) for each species and treatment combination.

Species	Treatment *	Min ± SD
*Ceramaster granularis*	Two same-sized individuals	21.0 ± 10.0
	Two differently sized individuals	40.0 ± 8.3
	Five differently sized individuals	45.0 ± 150.8
	*C. granularis* and *B. scalariforme* (short)	31.0 ± 17.0
	*C. granularis* and *B. scalariforme* (long)	17.5 ± 0.7
	*C. granularis*, *H. lisa*, and *B. scalariforme*	188.0 ± 341.7
	**Across all treatments**	**39.0 ± 161.7**
*Hippasteria phrygiana*	Two same-sized individuals	35.5 ± 11.3
	Two differently sized individuals	58.0
	Five differently sized individuals	50.0 ± 444.1
	**Across all treatments**	**45.5 ± 318.0**
*Henricia* *lisa*	Two same-sized individuals	26.0 ± 22.5
	*C. granularis* and *H. lisa* (long)	12.0 ± 8.5
	*C. granularis*, *H. lisa*, and *B. scalariforme*	65 ± 145.2
	**Across all treatments**	**22.0 ± 93.2**
*Buccinum scalariforme*	Two same-sized individuals	3.0 ± 22.8
	*C. granularis*, *H. lisa*, and *B. scalariforme*	103.0 ± 79.4
	**Across all treatments**	**48.5 ± 78.5**

* Treatment types that resulted in no positive response toward the food for a given species were omitted from the table for that species.

## Data Availability

Data are available upon request to the primary author.

## Supplementary Materials

The following supporting information can be downloaded at https://www.mdpi.com/article/10.3390/biology12060774/s1. Figure S1: Animal wet weight (g) to diameter/shell length (cm). Table S1: Size classes and mean sizes (± SD, *n* = 4–24) of focal species. Table S2: Mean percentage of total oral surface covered by podia (% ± SD) for each sea star species. Table S3: Behaviour category results (in % of total time ± SD) for prolonged-duration (18–23 h) trials. Table S4: Size classes used for each species in short-duration treatments. Video S1: Small *C. granularis* wedging under a large *C. granularis.* Video S2: Small *C. granularis* blocking a large *C. granularis* and four small *C. granularis* cooperatively feeding. Video S3: Large *C. granularis* pushing small *C. granularis* off food. Video S4: Large *H. phrygiana* skirting around small *H. phrygiana* to reach food. Video S5: Medium *H. phrygiana* pushing small *H. phrygiana* off food. Video S6: *H. lisa* traveling to food. Video S7: *H. lisa* tapping food. Video S8: *B. scalariforme* approaching food. Video S9: *C. granularis* wedging under *H. lisa.*
