# Supplementary material for: Intra- and Interspecific Foraging and Feeding Interactions in Three Sea Stars and a Gastropod from the Deep Sea
Source: Biology (Basel). 2023 May 26;12(6):774. doi: 10.3390/biology12060774 (PMC10295343; doi:10.3390/biology12060774)
Supplement: Supplementary file 1 [file biology-12-00774-s001.zip › Supplementary Figure S1.pdf]

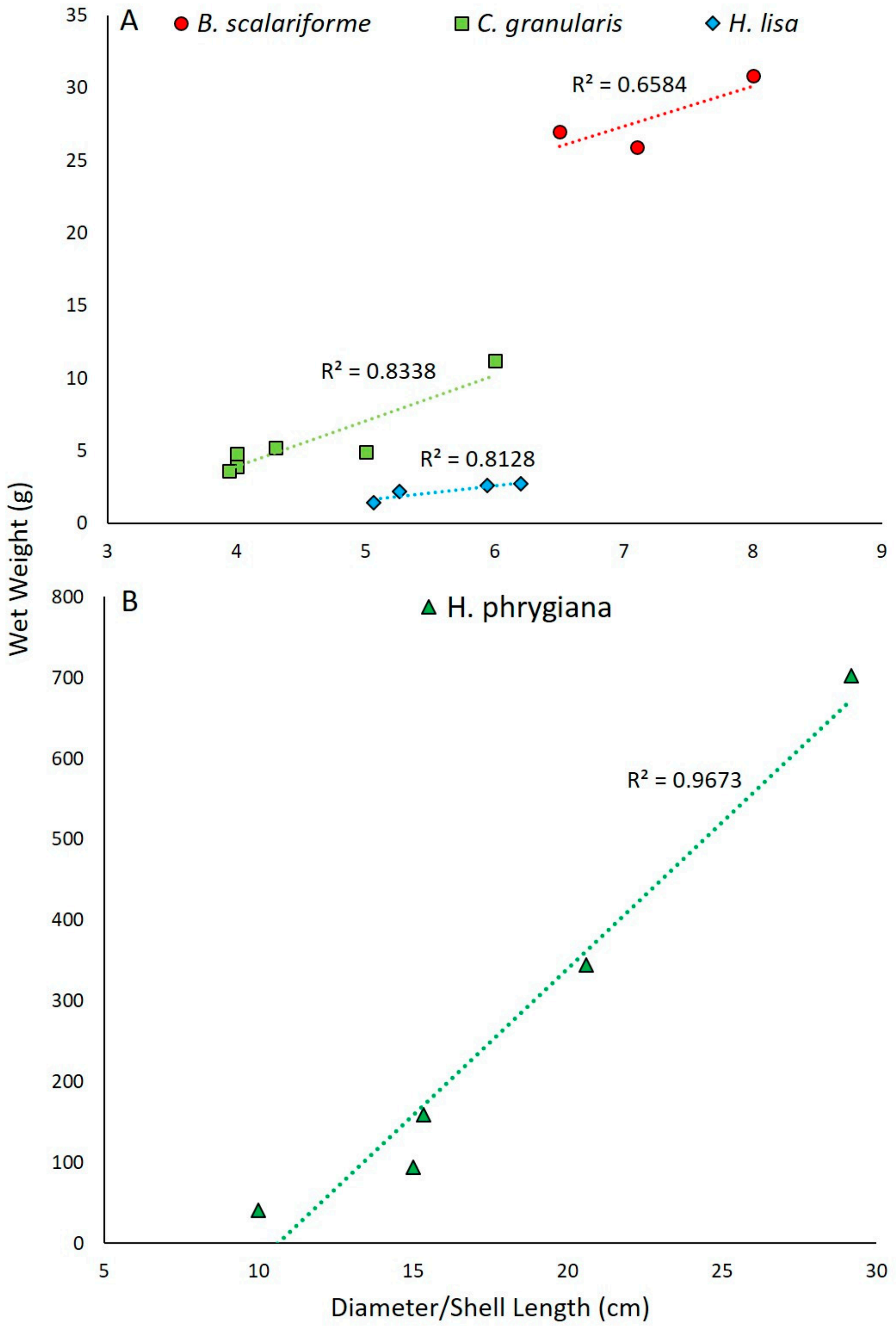

Figure S1: Animal wet weight (g) to diameter/shell length (cm) for (A) *Buccinum scalariforme*, *Ceramaster granularis*, *Henricia lisa*, and (B) *Hippasteria phrygiana* with R^2 values. Note the different X and Y scales in panels A and B. Results for *H. phrygiana* were graphed separately due to the very different body size scale of this species.
