# Supplementary material for: Intra- and Interspecific Foraging and Feeding Interactions in Three Sea Stars and a Gastropod from the Deep Sea
Source: Biology (Basel). 2023 May 26;12(6):774. doi: 10.3390/biology12060774 (PMC10295343; doi:10.3390/biology12060774)
Supplement: Supplementary file 1 [file biology-12-00774-s001.zip › Supplementary Table S1.pdf]

Table S1: Size classes and mean sizes (± SD, n = 4-24) of focal species used in different experimental treatments and food stimuli used for different treatments. Palatable foods were determined based on the results of Stuckless et al. (2021).

Species	Size Class	Diameter/Shell length (cm)	Treatments (duration)	Food Stimuli
Ceramaster granularis	Small	3.7 ± 0.2	Two *Ceramaster* different sizes (short)	Octopus
			Five *Ceramaster* (prolonged)	Octopus
	Medium	4.7 ± 1.1	Two *Ceramaster* same size (short)	Octopus
			Five *Ceramaster* (prolonged)	Octopus
			Ceramaster and *Henricia* (short and prolonged)	Sponge (short)<br>Octopus (prolonged)
			Ceramaster and *Buccinum* (short and prolonged)	Octopus (short)<br>Octopus (prolonged)
			Ceramaster, *Henricia*, and *Buccinum* (prolonged)	Octopus
	Large	6.9 ± 0.2	Two *Ceramaster* different sizes (short)	Octopus
			Five *Ceramaster* (prolonged)	Octopus
Hippasteria phrygiana	Small	11.9 ± 0.3	Two *Hippasteria* different sizes (short)	Cup coral
			Five *Hippasteria* (prolonged)	Cup coral
	Medium	15.3 ± 2.3	Two *Hippasteria* same size (short)	Cup coral
			Five *Hippasteria* (prolonged)	Cup coral
	Large	17.9 ± 1.3	Two *Hippasteria* different sizes (short)	Cup coral
			Five *Hippasteria* (prolonged)	Cup coral
Henricia lisa	N/A	5.3 ± 1.0	Two *Henricia* (short)	Sponge
			Ceramaster and *Henricia* (short and prolonged)	Sponge (short)<br>Octopus (prolonged)
			Ceramaster, *Henricia*, and *Buccinum* (prolonged)	Octopus
Buccinum scalariforme	N/A	6.5 ± 0.7	Two *Buccinum* (short)	Octopus
			Ceramaster and *Buccinum* (short and prolonged)	Octopus (short)<br>Octopus (prolonged)
			Ceramaster, *Henricia*, and *Buccinum* (prolonged)	Octopus
