# Supplementary material for: Intra- and Interspecific Foraging and Feeding Interactions in Three Sea Stars and a Gastropod from the Deep Sea
Source: Biology (Basel). 2023 May 26;12(6):774. doi: 10.3390/biology12060774 (PMC10295343; doi:10.3390/biology12060774)
Supplement: Supplementary file 1 [file biology-12-00774-s001.zip › Supplementary Table S2.pdf]

Table S2: Mean percentage of total oral surface covered by podia (% ± SD) for each sea star species as determined through photo analysis in ImageJ (n = 4-6)

**Species**	**Surface Covered by Podia (Mean% ± SD)**
Ceramaster granularis	19.6 ± 2.3
Hippasteria phrygiana	25.4 ± 0.9
Henricia lisa	28.5 ± 4.6
