# Supplementary material for: Intra- and Interspecific Foraging and Feeding Interactions in Three Sea Stars and a Gastropod from the Deep Sea
Source: Biology (Basel). 2023 May 26;12(6):774. doi: 10.3390/biology12060774 (PMC10295343; doi:10.3390/biology12060774)
Supplement: Supplementary file 1 [file biology-12-00774-s001.zip › Supplementary Table S3.pdf]

Table S3: Behaviour category results (in % of total time ± SD) for prolonged duration (18-23 h) trials for each species and size class, one measurement per hour for each trial (see text for description of behaviour categories).

Species	Food	Size/Species (n)	Behaviour	Treatment	% of Time (± SD)
Ceramaster granularis	Octopus	Small (4)	Immobile	Experimental	28.2 ± 35.2
				Control	14.9 ± 17.2
			Mobile	Experimental	21.4 ± 22.2
				Control	39.9 ± 29.6
			Contact Animal	Experimental	1.3 ± 2.5
				Control	2.3 ± 4.6
			Contact Stimulus	Experimental	0 ± 0
				Control	27.1 ± 45.9
			Feeding	Experimental	47.9 ± 55.4
				Control	0 ± 0
			Unknown	Experimental	1.3 ± 2.5
				Control	15.9 ± 31.8
		Medium (12)	Immobile	Experimental	25.4 ± 28.0
				Control	49.0 ± 37.8
			Mobile	Experimental	34.7 ± 31.2
				Control	35.0 ± 27.5
			Contact Animal	Experimental	0.8 ± 2.0
				Control	0.8 ± 2.6
			Contact Stimulus	Experimental	0 ± 0
				Control	0 ± 0
			Feeding	Experimental	36.1 ± 40.8
				Control	0 ± 0
			Unknown	Experimental	3.5 ± 6.5
				Control	15.2 ± 28.8
		Large (4)	Immobile	Experimental	11.7 ±17.8
				Control	68.9 ± 15.5
			Mobile	Experimental	62.5 ± 40.8
				Control	26.8 ± 8.5
			Contact Animal	Experimental	0 ± 0
				Control	0 ± 0
			Contact Stimulus	Experimental	0 ± 0
				Control	0 ± 0
			Feeding	Experimental	23.8 ± 47.5
				Control	0 ± 0
			Unknown	Experimental	4.4 ± 6.2
				Control	4.4 ± 8.7
Hippasteria phrygiana	Cup Coral	Small (4)	Immobile	Experimental	49.1 ± 47.6
				Control	61.9 ± 22.8
			Mobile	Experimental	44.3 ± 44.2
				Control	38.1 ± 24.6
			Contact Animal	Experimental	2.2 ± 2.5
				Control	0 ± 0
			Contact Stimulus	Experimental	0 ± 0
				Control	0 ± 0
			Feeding	Experimental	1.1 ± 2.2
				Control	0 ± 0
			Unknown	Experimental	3.3 ± 4.2
				Control	0 ± 0
		Medium	Immobile	Experimental	38.3 ± 32.4

		(12)		Control	46.1 ± 36.7
			Mobile	Experimental	41.8 ± 30.5
				Control	41.8 ± 31.3
			Contact Animal	Experimental	3.3 ± 5.9
				Control	10.6 ± 20.9
			Contact Stimulus	Experimental	0 ± 0
				Control	0 ± 0
			Feeding	Experimental	15.6 ± 36.4
				Control	0 ± 0
			Unknown	Experimental	0.7 ± 2.5
				Control	1.5 ± 4.02
		Large (4)	Immobile	Experimental	48.5 ± 32.6
				Control	30.9 ± 32.4
			Mobile	Experimental	27.7 ± 16.0
				Control	66.7 ± 31.7
			Contact Animal	Experimental	0 ± 0
				Control	1.2 ± 2.4
			Contact Stimulus	Experimental	0 ± 0
				Control	0 ± 0
			Feeding	Experimental	23.9 ± 47.7
				Control	0 ± 0
			Unknown	Experimental	0 ± 0
				Control	0 ± 0
**Ceramaster granularis* + *Henricia lisa*	Sponge	*C. granularis* (2)	Immobile	Experimental	52.2 ± 24.6
				Control	15.2 ± 9.2
			Mobile	Experimental	43.5 ± 18.5
				Control	76.1 ± 9.2
			Contact Animal	Experimental	0 ± 0
				Control	0 ± 0
			Contact Stimulus	Experimental	0 ± 0
				Control	0 ± 0
			Feeding	Experimental	0 ± 0
				Control	0 ± 0
			Unknown	Experimental	4.4 ± 6.2
				Control	8.7 ± 0
		H. lisa (2)	Immobile	Experimental	0 ± 0
				Control	21.7 ± 30.7
			Mobile	Experimental	2.2 ± 3.1
				Control	58.7 ± 46.1
			Contact Animal	Experimental	0 ± 0
				Control	0 ± 0
			Contact Stimulus	Experimental	0 ± 0
				Control	0 ± 0
			Feeding	Experimental	97.8 ± 3.1
				Control	0 ± 0
			Unknown	Experimental	0 ± 0
				Control	19.6 ± 15.4
**Ceramaster granularis* + *Buccinum scalariforme*	Octopus	*C. granularis* (2)	Immobile	Experimental	0 ± 0
				Control	43.9 ± 49.8
			Mobile	Experimental	4.3 ± 0.1
				Control	34.3 ± 19.1
			Contact Animal	Experimental	0 ± 0
				Control	4.4 ± 6.2
			Contact Stimulus	Experimental	0 ± 0
				Control	0 ± 0
			Feeding	Experimental	95.7 ± 0.1

				Control	0 ± 0
			Unknown	Experimental	0 ± 0
				Control	17.4 ± 24.6
		B. scalariforme (2)	Immobile	Experimental	65.7 ± 19.1
				Control	47.6 ± 49.6
			Mobile	Experimental	34.3 ± 19.1
				Control	27.3 ± 26.3
			Contact Animal	Experimental	0 ± 0
				Control	4.4 ± 6.2
			Contact Stimulus	Experimental	0 ± 0
				Control	0 ± 0
			Feeding	Experimental	0 ± 0
				Control	0 ± 0
			Unknown	Experimental	0 ± 0
				Control	20.8 ± 29.5
Ceramaster granularis + *Henricia lisa* + *Buccinum scalariforme*	Octopus	*C. granularis* (4)	Immobile	Experimental	5.0 ± 7.1
				Control	29.1 ± 36.7
			Mobile	Experimental	36.3 ± 45.7
				Control	50.3 ± 31.8
			Contact Animal	Experimental	1.3 ± 2.5
				Control	5.7 ± 8.6
			Contact Stimulus	Experimental	0 ± 0
				Control	0 ± 0
			Feeding	Experimental	57.5 ± 43.5
				Control	0 ± 0
			Unknown	Experimental	0 ± 0
				Control	14.9 ± 23.8
		H. lisa (4)	Immobile	Experimental	0 ± 0
				Control	7.5 ± 15.0
			Mobile	Experimental	9.5 ± 8.5
				Control	50.8 ± 37.6
			Contact Animal	Experimental	0 ± 0
				Control	2.4 ± 4.8
			Contact Stimulus	Experimental	0 ± 0
				Control	23.9 ± 47.7
			Feeding	Experimental	69.2 ± 46.8
				Control	0 ± 0
			Unknown	Experimental	21.3 ± 42.5
				Control	15.5 ± 31.0
		B. scalariforme (4)	Immobile	Experimental	49.5 ± 38.2
				Control	38.8 ± 26.0
			Mobile	Experimental	42.1 ± 43.3
				Control	35.9 ± 9.9
			Contact Animal	Experimental	0 ± 0
				Control	5.7 ± 8.6
			Contact Stimulus	Experimental	0 ± 0
				Control	0 ± 0
			Feeding	Experimental	8.5 ± 11.3
				Control	0 ± 0
			Unknown	Experimental	0 ± 0
				Control	19.6 ± 24.3

*Indicates experimental trials with two replicates instead of four.
