# Supplementary material for: Intra- and Interspecific Foraging and Feeding Interactions in Three Sea Stars and a Gastropod from the Deep Sea
Source: Biology (Basel). 2023 May 26;12(6):774. doi: 10.3390/biology12060774 (PMC10295343; doi:10.3390/biology12060774)
Supplement: Supplementary file 1 [file biology-12-00774-s001.zip › Supplementary Table S4.pdf]

Table S4: Size classes used for each species in short duration treatments with distance traveled, mean speed, and maximum speed provided as mean ± SD for positive, negative, and control trials.

Species	Treatment (Food)	Size Class	Variable	Score*	Value (n)
Ceramaster granularis	Same size (Octopus)	N/A	Distance (cm)	Positive	9.0 ± 5.2 (4)
				Negative	6.1 ± 5.4 (4)
				Control	5.8 ± 3.2 (8)
			Mean Speed (cm min^{-1})	Positive	0.2 ± 0.1 (4)
				Negative	0.1 ± 0.1 (4)
				Control	0.1 ± 0.1 (8)
			Maximum Speed (cm min^{-1})	Positive	0.9 ± 0.5 (4)
				Negative	0.6 ± 0.4 (4)
				Control	0.8 ± 0.2 (8)
	Different sizes (Octopus)	Small	Distance (cm)	Positive	5.3 ± 2.5 (2)
				Negative	0.6 ± 0.6 (2)
				Control	1.4 ± 1.5 (4)
			Mean Speed (cm min^{-1})	Positive	0.1 ± 0.0 (2)
				Negative	0.0 ± 0.0 (2)
				Control	0.0 ± 0.0 (4)
			Maximum Speed (cm min^{-1})	Positive	0.6 ± 0.1 (2)
				Negative	0.3 ± 0.1 (2)
				Control	0.3 ± 0.1 (4)
		Large	Distance (cm)	Positive	7.6 (1)
				Negative	0.1 ± 0.1 (3)
				Control	1.5 ± 2.1 (4)
			Mean Speed (cm min^{-1})	Positive	0.2 (1)
				Negative	0.0 ± 0.0 (3)
				Control	0.0 ± 0.1 (4)
			Maximum Speed (cm min^{-1})	Positive	0.8 (1)
				Negative	0.1 ± 0.1 (3)
				Control	0.2 ± 0.2 (4)
Hippasteria phrygiana	Same size (Cup coral)	N/A	Distance (cm)	Positive	22.7 ± 4.2 (4)
				Negative	20.1 ± 13.6 (4)
				Control	10.5 ± 11.2 (8)
			Mean Speed (cm min^{-1})	Positive	0.4 ± 0.0 (4)
				Negative	0.3 ± 0.2 (4)
				Control	0.2 ± 0.2 (8)
			Maximum Speed (cm min^{-1})	Positive	1.3 ± 0.2 (4)
				Negative	1.2 ± 0.4 (4)
				Control	0.8 ± 0.5 (8)
	Different sizes (Cup coral)	Small	Distance (cm)	Positive	(0)
				Negative	1.4 ± 1.3 (4)
				Control	1.1 ± 2.0 (4)
			Mean Speed	Positive	(0)

			(cm min^{-1})	Negative	0.0 ± 0.0 (4)
				Control	0.0 ± 0.1 (4)
			Maximum Speed	Positive	(0)
			(cm min^{-1})	Negative	0.4 ± 0.1 (4)
				Control	0.3 ± 0.3(4)
		Large	Distance	Positive	14.9 (1)
			(cm)	Negative	2.3 ± 1.6 (3)
				Control	16.6 ± 12.0 (4)
			Mean Speed	Positive	0.2 (1)
			(cm min^{-1})	Negative	0.1 ± 0.1 (3)
				Control	0.4 ± 0.2 (4)
			Maximum Speed	Positive	1.1 (1)
			(cm min^{-1})	Negative	0.5 ± 0.3 (3)
				Control	1.4 ± 0.8 (4)
Henricia	Two	N/A	Distance	Positive	9.5 ± 5.3 (3)
lisa	individuals		(cm)	Negative	14.0 ± 11.3 (5)
	(Sponge)			Control	14.8 ± 9.5 (8)
			Mean Speed	Positive	0.2 ± 0.0 (3)
			(cm min^{-1})	Negative	0.3 ± 0.2 (5)
				Control	0.3 ± 0.2 (8)
			Maximum Speed	Positive	0.8 ± 0.2 (3)
			(cm min^{-1})	Negative	1.0 ± 0.4 (5)
				Control	0.8 ± 0.3 (8)
Buccinum	Two	N/A	Distance	Positive	67.5 ± 105.2 (3)
scalariforme	individuals		(cm)	Negative	104.3 ± 82.0 (5)
	(Octopus)			Control	84.7 ± 80.9 (8)
			Mean Speed	Positive	1.6 ± 1.5 (3)
			(cm min^{-1})	Negative	3.2 ± 1.5 (5)
				Control	3.4 ± 3.0 (8)
			Maximum Speed	Positive	6.0 ± 4.6 (3)
			(cm min^{-1})	Negative	9.6 ± 1.1 (5)
				Control	8.5 ± 4.0 (8)
Ceramaster	One	Medium	Distance	Positive	(0)
granularis	individual	(*C.*	(cm)	Negative	10.2 ± 5.8 (4)
+	of each	*granularis*)		Control	6.4 ± 4.5 (4)
Henricia	(Sponge)		Mean Speed	Positive	(0)
lisa			(cm min^{-1})	Negative	0.2 ± 0.1 (4)
				Control	0.1 ± 0.1 (4)
			Maximum Speed	Positive	(0)
			(cm min^{-1})	Negative	0.7 ± 0.2 (4)
				Control	0.7 ± 0.2 (4)
		N/A	Distance	Positive	(0)
		(*H. lisa*)	(cm)	Negative	42.1 ± 17.8 (4)
				Control	18.0 ± 6.1 (4)
			Mean Speed	Positive	(0)
			(cm min^{-1})	Negative	0.7 ± 0.2 (4)

				Control	0.3 ± 0.1 (4)
			Maximum Speed (cm min^{-1})	Positive	(0)
				Negative	2.2 ± 0.6 (4)
				Control	1.3 ± 0.3 (4)
Ceramaster granularis + *Buccinum scalariforme*	One individual of each (Octopus)	Medium (*C. granularis*)	Distance (cm)	Positive	10.5 ± 2.6 (2)
				Negative	28.7 ± 30.0 (2)
				Control	12.8 ± 4.4 (4)
			Mean Speed (cm min^{-1})	Positive	0.2 ± 0.1 (2)
				Negative	0.5 ± 0.5 (2)
				Control	0.2 ± 0.1 (4)
			Maximum Speed (cm min^{-1})	Positive	0.8 ± 0.1 (2)
				Negative	1.7 ± 1.4 (2)
				Control	1.3 ± 0.4 (4)
		N/A (*B. scalariforme*)	Distance (cm)	Positive	(0)
				Negative	190.1 ± 72.1 (4)
				Control	145.3 ± 127.9 (4)
			Mean Speed (cm min^{-1})	Positive	(0)
				Negative	3.5 ± 0.8 (4)
				Control	2.9 ± 2.4 (4)
			Maximum Speed (cm min^{-1})	Positive	(0)
				Negative	12.3 ± 2.1 (4)
				Control	10.8 ± 6.2 (4)

*Positive trials resulted in feeding, negative trials did not result in feeding, all control trials were pooled.
